# Supplementary figures and images for: Protective mechanism of SIRT1 on Hcy‐induced atrial fibrosis mediated by TRPC3
Source: J Cell Mol Med. 2019 Nov 4;24(1):488–510. doi: 10.1111/jcmm.14757 (PMC6933351; doi:10.1111/jcmm.14757)

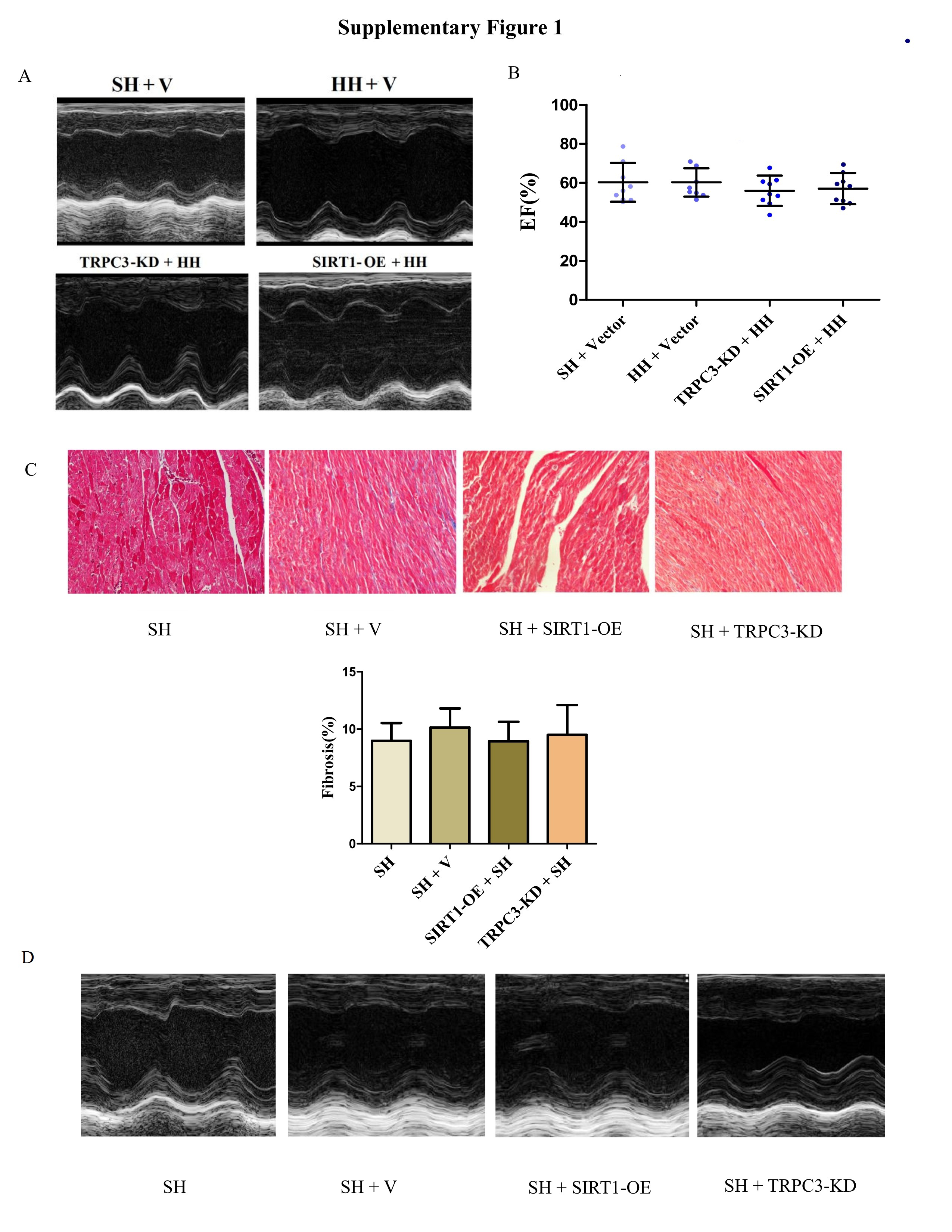

Supplement: Supplementary file 1 [file JCMM-24-488-s001.tif]

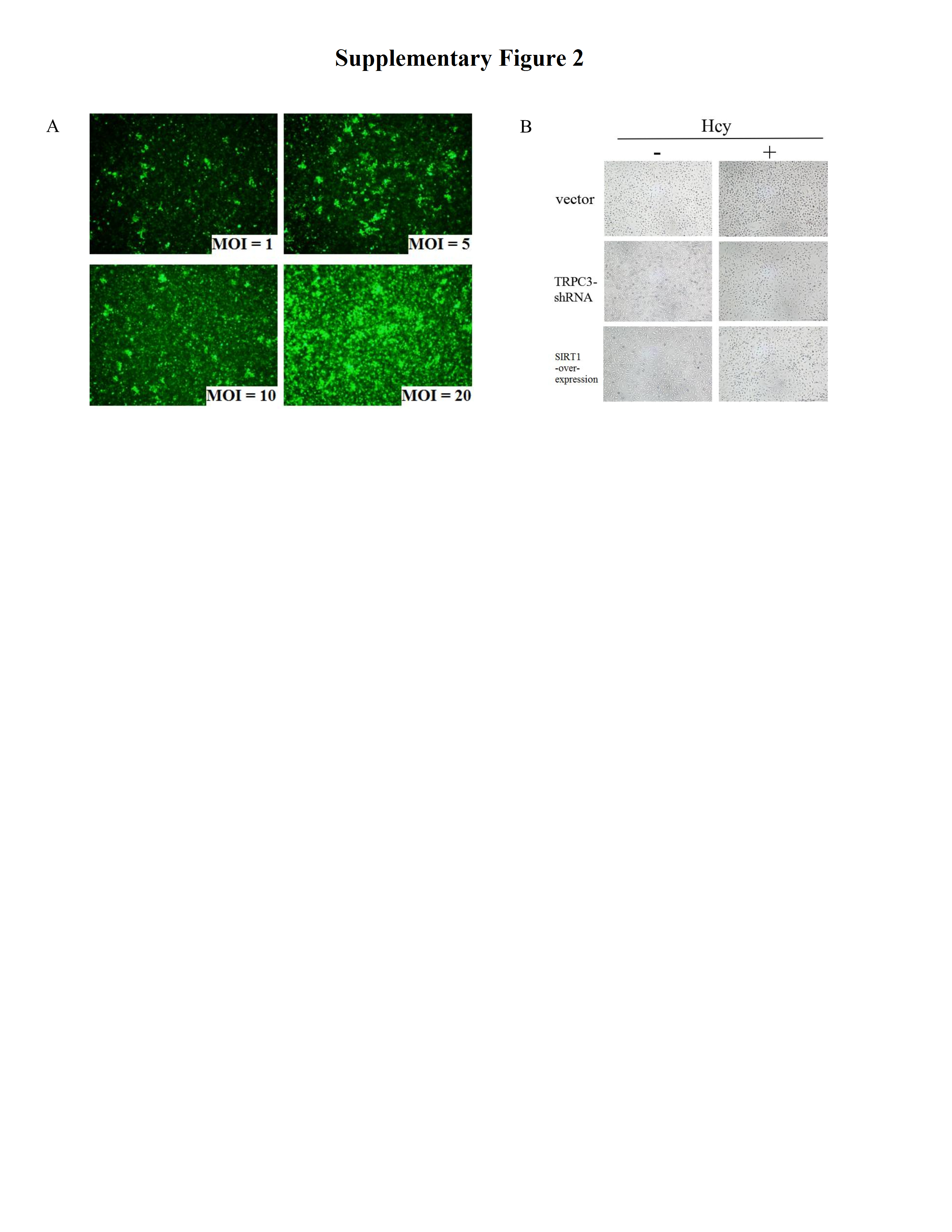

Supplement: Supplementary file 2 [file JCMM-24-488-s002.tif]

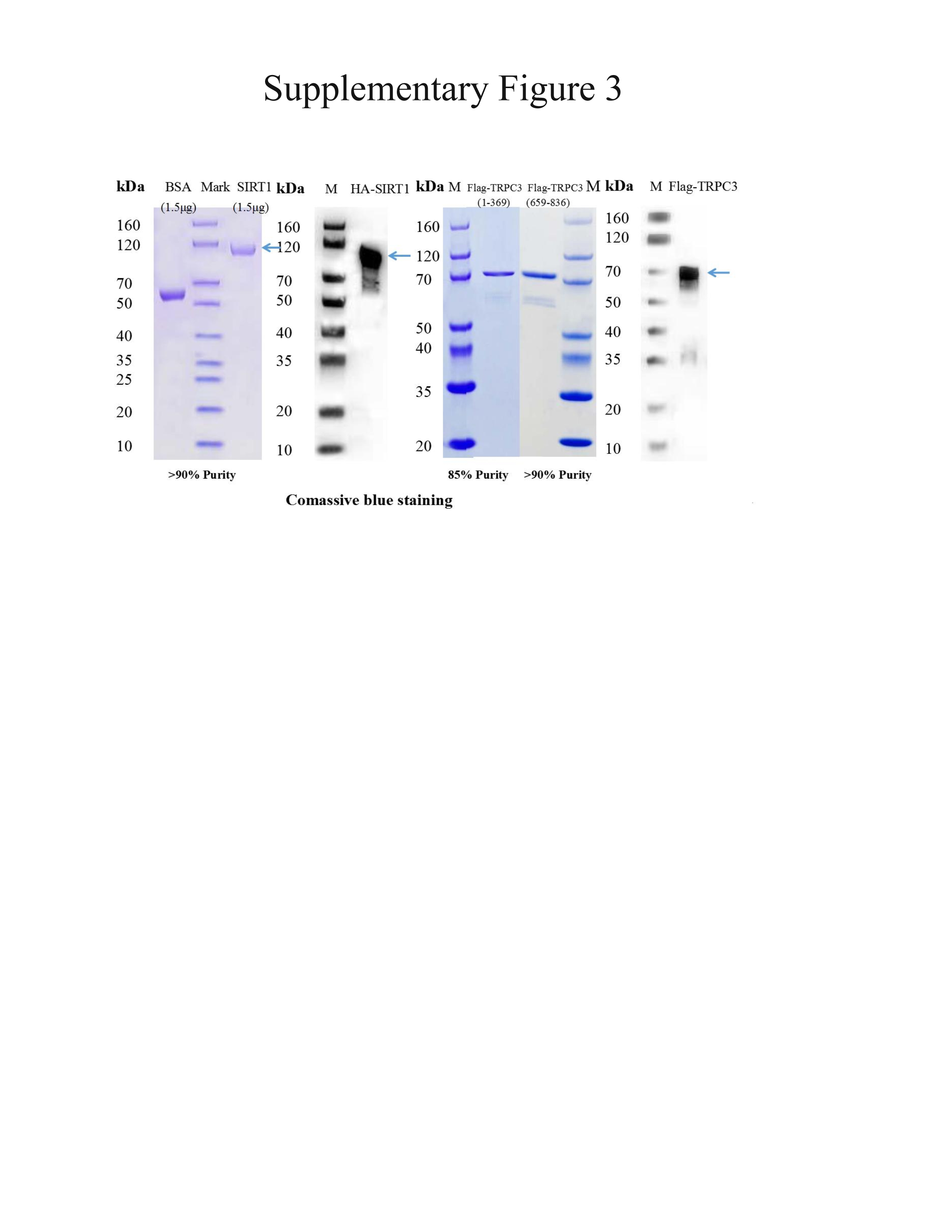

Supplement: Supplementary file 3 [file JCMM-24-488-s003.tif]
